# Editorial: Neuromodulation for pharmacoresistant epilepsy: from bench to bed

**DOI:** 10.3389/fneur.2024.1354897

**Published:** 2024-02-26

**Authors:** Tianfu Li, Jiahui Deng, Jiong Qin, Xiang-Ping Chu

**Affiliations:** ^1^Department of Neurology, Beijing Institute for Brain Disorders, Beijing Key Laboratory of Epilepsy Research, Sanbo Brain Hospital, Capital Medical University, Beijing, China; ^2^Department of Pediatrics, Peking University People's Hospital, Beijing, China; ^3^Departments of Biomedical Sciences and Anesthesiology, School of Medicine University of Missouri-Kansas City, Kansas City, KS, United States

**Keywords:** neuromodulation, pharmacoresistant epilepsy, drug-resistant epilepsy, deep brain stimulation, responsive neurostimulation, vagus nerve stimulation, transcranial magnetic stimulation, ultrasonic therapy

Epilepsy is a persistent neurological disorder that affects more than 70 million people worldwide. It is characterized by a long-lasting predisposition to recurrently generate epileptic seizures, as well as accompanying psychiatric and cognitive comorbidities (1). Currently, about one-third of all people with epilepsy was drug-resistant epilepsy. Resection of epileptogenic tissue to suppress the epileptic crisis remains the last resort in some drug-resistant patients. However, a large number of patients are not candidates for surgical resective therapy, facing unmet medical needs. Therefore, it is imperative to develop alternative therapies leading to seizure remission. Neuromodulation is one such alternative treatment. There are several neuromodulation methods, including invasive therapies that require an implantable device and electrodes—such as deep brain stimulation (DBS), responsive neurostimulation (RNS), and vagus nerve stimulation (VNS)—and non-invasive approaches, such as transcranial magnetic stimulation (TMS) and ultrasonic therapy (2). Patients' selection, optimal anatomical targets, best stimulation parameters, prediction of neuromodulation therapy outcome, and understanding the underlying mechanisms are currently challenging.

Regarding these, we are pleased to present the collection of papers in this Research Topic, *Neuromodulation for pharmacoresistant epilepsy: from bench to bed*. This Research Topic includes 10 articles covering from clinical to basic research. It consists of six original articles, two study protocols, one original research review, and one brief research report.

DBS of the anterior nucleus of the thalamus (ANT-DBS) is currently approved for the treatment of refractory focal epilepsy. Based on a single central clinical result, the original clinical research article by Yan et al. demonstrated that ANT-DBS was effective for patients with either temporal lobe epilepsy or extratemporal lobe epilepsy. In addition, DBS of subthalamic nucleus could potentially serve as an effective therapy for patients with motor seizures, particularly when the epileptogenic zone overlaps with the sensorimotor cortex. Centromedian nucleus (CMN) and pulvinar nucleus could be regarded as modulating targets for patients with Lennox-Gastaut syndrome-like epilepsy or occipital lobe epilepsy, respectively. Another single center research article by Dague et al. presented the possible undesired psychiatric side effects and the short/long-term effects on patients' neuropsychological assessment. To clarify the possible reason of these side effects might help to improve the clinical operation and postoperative programing for ANT-DBS.

The RNS system delivers electrical stimulation on detection of ictal intracranial EEG for medically refractory focal-onset epilepsy. The original clinical research article by Fields et al. was conducted in a multicenter retrospective study of patients treated in the thalamus RNS from seven epilepsy centers in the United States. The article suggested that RNS treatment in either the ANT or CMN of thalamus was safe and effective in reducing seizure frequency and improving quality of life in patients with different seizure types. The single center research article by Owens et al. suggested that preoperative stereoelectroencephalography (sEEG) was helpful to increase the positive response rates of RNS in patients.

VNS is regarded as a minimally invasive, peripheral method for modulating epileptic networks. The original clinical research article by Guo et al. demonstrated the efficacy and safety of VNS in the treatment of pharmacoresistant epilepsy secondary to encephalomalacia. Moreover, the article suggested the potential predictors of VNS effectiveness, including seizure onset age (>18 years old), unilateral interictal epileptic discharges, and unilateral encephalomalacia on MRI. The original clinical research article by Iimura et al. determined that generalized seizure was most responsive to VNS and investigated the preventive effect of VNS on status epilepticus (SE) recurrence. The study protocol article by Verner et al. described a prospective, open-label, multicenter phase I clinical trial designed to evaluate the potential safety and efficacy of high frequency bursts of stimulation known as “Microburst VNS” (μVNS) in patients with refractory focal and generalized epilepsies. This protocol also utilized an investigational, fMRI-guided titration approach that allows for personalized dosing of μVNS based on the thalamic blood-oxygen-level-dependent signal.

Repetitive TMS (rTMS), as a focal, non-invasive method, shows potential for applications in epilepsy. The original clinical research article by Yang et al. described the favorable outcomes after low-frequency rTMS ( ≤ 1 Hz) in patients with self-limited epilepsy with centrotemporal spikes (SeLECTS) with electrical status epilepticus in sleep (ESES). By analyzing the aperiodic offset and slope of EEG data, they determined the impact of rTMS on the excitation–inhibition imbalance in the patients' brains. The findings suggested that rTMS might lead to a reduction in firing rates in neuronal populations, particularly at the site of stimulation.

Therapeutic focused ultrasound (FUS) is a noninvasive brain stimulation treatment that targets a specific part of the brain by using energy in the form of acoustic waves beyond the range of human hearing.

The review by Cornelssen et al. discussed preclinical and clinical FUS studies to treat seizures and presented investigated potential applications of FUS for targeted drug delivery to the seizure foci. Additionally, they summarized its effective parameters and analyzed the future directions and constraints of FUS in the treatment of epilepsy.

Cognitive dysfunction is prevalent in epilepsy which may have a significant impact on social functioning and quality of life. The study protocol article by Puteikis et al. described a randomized waitlist-controlled trial of cognitive rehabilitation in epilepsy (CoRE) with the aim of improving both quality of life and cognitive functioning in a mixed sample of people with epilepsy (PWE). Through the endeavor, neuropsychological evaluation experience would be further translated into non-invasive add-on rehabilitation treatments that addressed PWE's bothersome cognitive difficulties.

## Author contributions

TL: Writing—original draft, Writing—review & editing. JD: Writing—original draft. JQ: Writing—review & editing. X-PC: Writing—review & editing.
